# Amino acid derangements in adults with severe falciparum malaria

**DOI:** 10.1038/s41598-019-43044-6

**Published:** 2019-04-29

**Authors:** Stije J. Leopold, Siribha Apinan, Aniruddha Ghose, Hugh W. Kingston, Katherine A. Plewes, Amir Hossain, Asok Kumar Dutta, Sujat Paul, Anupam Barua, Abdus Sattar, Nicholas P. J. Day, Joel Tarning, Markus Winterberg, Nicholas J. White, Arjen M. Dondorp

**Affiliations:** 1 Mahidol-Oxford Tropical Medicine Research Unit, Faculty of Tropical Medicine, Mahidol University, Bangkok, Thailand; 2 Centre for Tropical Medicine and Global Health, Nuffield Department of Medicine, University of Oxford, Oxford, United Kingdom; 3 Department of Internal Medicine, Chittagong Medical College Hospital, Chittagong, Bangladesh

**Keywords:** Translational research, Infection

## Abstract

Amino acid derangements are common in severe falciparum malaria and have been associated with endothelial dysfunction (L-arginine), metabolic acidosis (alanine and lactate), and disease severity (phenylalanine and tryptophan metabolites). Whether these amino acid perturbations reflect isolated pathogenic mechanisms or if they are part of overall changes in amino acid metabolism is unclear. To investigate this, we prospectively simultaneously quantified a broad range of plasma free amino acids (PFAA) using HPLC-MRM-Mass spectrometry in relation to presenting symptoms in adults with severe malaria (n = 88), septicaemia (n = 88), uncomplicated malaria (n = 71), and healthy controls (n = 48) from Bangladesh. The total plasma concentration of measured amino acids was significantly reduced in each of the patient groups when compared to normal levels observed in healthy local controls: uncomplicated malaria −54%, severe malaria −23%, and sepsis −32%, (p = <0.001). Inspection of amino acid profiles revealed that in each group the majority of amino acids were below normal levels, except for phenylalanine. Among patients with severe malaria, L-lactate was strongly associated with an increase of the total amino acid concentration, likely because this reflects tissue hypoxia. Our data confirm previously described amino acid abnormalities, likely resulting from overall changes in the concentration of PFAA.

## Introduction

Severe malaria, caused by infection with *Plasmodium falciparum*, remains an important contributor to premature death in endemic countries^1^. The World Health Organisation estimated 445,000 patients died from malaria infection in 2016, predominantly in sub-Saharan Africa^2^. As parenteral artesunate is now established as the best drug to treat severe falciparum malaria^3,4^, further gains in reducing case fatality may be achieved by the development of adjunctive therapies. To develop new adjunctive therapies for severe malaria increased understanding is required of the underlying pathophysiology of life-threatening complications.

The pathophysiology of severe falciparum malaria is linked to derangements in amino acid metabolism. Key biochemical abnormalities in falciparum malaria consist of L-arginine deficiency, associated with endothelial dysfunction^5,6^, elevated L-lactate and alanine, associated with metabolic acidosis^7,8^, elevated phenylalanine^9–12^ and increased tryptophan metabolites^13,14^, both associated with disease severity, and low glutamine, a modulation of oxidative stress^15^. Besides these, there are multiple other amino acid abnormalities described previously, such as low ornithine, lysine, and histidine in cases with falciparum malaria^10^. Prior studies have described associations between specific amino acid derangements and clinical outcomes, and new adjunctive therapies are being developed to correct amino acid disorders. However, whether these amino acid derangements are isolated pathogenic mechanisms or if they are part of overall changes in amino acid metabolism is unclear.

The plasma concentration of free amino acids reflects a balance between tissue amino acid utilisation, and intestinal absorption, protein turnover, and *de novo* synthesis, which are under hormonal regulation and modulated by the bioavailability of cofactors and disease^16^. During critical illness, the circulating concentration of PFAA may be affected by an energy deficit, hypoxia, and underlying malnutrition^17–20^. An acute energy deficit can develop because of reduced intake (coma) and elevated energy requirements (infection), associated with increased hepatic extraction of amino acids from the bloodstream^17,19,21^. Tissue hypoxia increases cellular energy requirements because of incomplete anaerobic oxidation of glucose, and increases protein breakdown to allow amino acids to be converted to sugars or other energy substrates through oxidative deamination^16^. Underlying malnutrition may predispose and aggravate a negative energy balance^20^.

During malaria infection, *Plasmodium falciparum* parasites adhere to the vascular endothelium of the microcirculation, where they sequester and compromise microvascular blood flow. Patterns of an obstructed microcirculation in patients with severe falciparum malaria correlate strongly with hyperlactataemia^22,23^. Abnormal L-lactate-to-pyruvate ratios in malaria suggest hyperlactataemia derives from anaerobic glycolysis^7^, and that L-lactate is a marker of underlying tissue hypoxia. In severe falciparum malaria tissue hypoxia might increase the overall concentration of PFAA.

To determine the interrelationships between several individual amino acid disorders in patients with malaria we simultaneously quantified and profiled a broad range of amino acids in plasma by high-performance multiple-reaction-monitoring mass-spectrometry (HPLC-MRM-MS). We hypothesised that amino acid derangements in patients with malaria are not isolated disorders but that they follow overall changes in amino acid metabolism, characterised by an overall reduction of the concentration of PFAA in the context of infection. While in severe falciparum malaria, we postulated, L-lactate is related to an increase in the total concentration of PFAA. These metabolomic studies might help improve the understanding of the pathogenesis of amino acid derangements in severe malaria, with potential implications for their adjunctive treatment.

## Participants

A total of 295 participants were consecutively enrolled in a prospective observational study in Chittagong Bangladesh, between 2014–2017, including adults with severe malaria (n = 88), septicaemia (n = 88), uncomplicated malaria (n = 71), and healthy controls (n = 48). The overall case fatality rate of severe falciparum malaria was 30%, and that of sepsis was 23%. Baseline characteristics of the study population are described in Table 1.

**Table 1:** Baseline characteristics in patients and healthy controls (n = 295) enrolled in a prospective observational study on amino acid derangements in severe falciparum malaria in Bangladesh.

	Severe Malaria (n = 88)		Uncomplicated Malaria (n = 71)		SIRS/Sepsis (n = 88)		Healthy Controls (n = 48)		p value
Age (years)	28	(19 to 40)	29	(22 to 45)	35	(23 to 54)	29	(23.5 to 35)	0.016
Female sex	32	36	19	27	37	42	19	40	0.172
BMI	21	(19 to 24)	21	(19 to 24)	22	(20 to 25)	24	(21 to 26)	0.004
Duration of illness (days)	8	(7 to 10)	8	(6 to 11)	9	(5 to 14)	—	—	0.01
Anorexia before enrolment (days)	8	(6 to 10)	7	(5 to 11)	7	(4 to 14)	—	—	0.025
Mortality	26	(30%)	0	—	20	(23%)	0	—	<0.001
**Clinical examination**									
Temperature (°C)	38.3	(37.3 to 39.1)	37.4	(37 to 38.4)	38.4	(38 to 38.9)	36.6	(36.3 to 36.8)	<0.001
Coma depth*	9	(7 to 14)	15	(15 to 15)	15	(11 to 15)	15	(15 to 15)	<0.001
Pulse rate (per minute)	110	(96 to 130)	98	(89 to 109)	107	(92 to 120)	82	(73 to 88)	<0.001
Respiratory rate (per minute)	34	(28 to 42)	24	(24 to 28)	32	(26 to 39)	18	(16 to 20)	<0.001
Oxygen saturation (%)	96	(95 to 97)	97	(96 to 98)	96	(94 to 98)	98	(97 to 98)	<0.001
Mean arterial blood pressure (mm Hg)	81	(72 to 88)	78	(71 to 84)	85	(77 to 98)	93	(89 to 97)	<0.001
**Laboratory assessments**									
Parasitaemia (parasites per µL)^†^	46705	(25541 to 85406)	4321	(1901 to 9819)	0	—	0	—	<0.001
*Pf*HPR2 (ng/dL)^†^	4165	(3101 to 5594)	640	(426 to 962)	0	—	0	—	<0.001
Glucose (mmol/L)	6.6	(5.2 to 8.3)	6.6	(5.6 to 7.9)	7	(6.1 to 9.4)	5.8	(5.2 to 6.3)	<0.001
Sodium (mmol/L	135	(131 to 139)	135	(132 to 138)	134	(130 to 138)	140	(139 to 141)	<0.001
Potassium (mmol/L)	3.9	(3.5 to 4.3)	3.4	(3.3 to 3.6)	3.5	(3.2 to 3.9)	3.7	(3.5 to 3.9)	<0.001
Chloride (mmol/L)	105	(100 to 110)	103	(98.5 to 106)	98.5	(94.8 to 103)	103	(102 to 105)	<0.001
Creatinine (µmol/L)	122	(82 to 317)	82	(65 to 103)	75.5	(57.5 to 113.3)	72.5	(57.8 to 81.3)	<0.001
Blood urea nitrogen (mmol/L)	40	(25 to 78)	16	(11 to 25)	11.5	(7 to 21.3)	8	(5.8 to 10)	<0.001
Haemoglobin (g/L)	83	(64 to 108)	97	(78 to 115.8)	118	(104 to 133)	134	(120 to 148)	<0.001
White blood cells (x 10^3^/µL)	9.1	(6.6 to 12.8)	6	(4.6 to 7.9)	11.1	(7 to 15)	7.7	(6.3 to 8.8)	<0.001
Platelets (x 10^3^/µL)	32	(19 to 47)	47	(35 to 96)	200	(121 to 277)	228	(191 to 289)	<0.001
pH	7.39	(7.35 to 7.44)	7.43	(7.39 to 7.46)	7.42	(7.38 to 7.45)	7.37	(7.35 to 7.39)	<0.001
pCO2 (mm Hg)	31	(27 to 34)	35	(31 to 36)	34	(31 to 39)	47	(42 to 52)	<0.001
HCO3- (mmol/L)	18.8	(16.3 to 21.1)	22.5	(20.7 to 25)	22.9	(20.1 to 25.1)	26.7	(24.9 to 28.6)	<0.001
Plasma SBD (mmol/L)	6	(3 to 10)	1.5	(−1 to 4)	1	(−0.3 to 5)	−1	(−3 to 0)	<0.001
Anion gap (mmol/L)	16	(14 to 18.8)	15	(13 to 16)	17	(15 to 19)	16	(14 to 17)	<0.001
L-lactate (mmol/L)	3.1	(2 to 4.8)	1.5	(1.1 to 1.8)	1.5	(1.1 to 2)	1.3	(1.1 to 1.6)	<0.001

Data are number (%) or median (IQR) unless otherwise indicated. BMI = body mass index. *Pf*HRP2 = Plasma *P. falciparum* Histidine Rich Protein 2. pCO2 = partial pressure of carbon dioxide. HCO3- = bicarbonate. SBD = standard base deficit. *Depth of coma was assessed by Glasgow coma scale (3–15). ^†^Geometric mean, 95% geometric confidence interval.

## Plasma free amino acids

Using HPLC-MRM-MS, we measured a total of 30 amino acids in plasma of study participants. Amino acids were excluded from the final analysis if they were detected in only a small number of cases (n < 50). We excluded 1-methylhistidine, alpha-aminoadipic acid, threonine, homocysteine, sarcosine, thiaproline, alpha-aminopimelic acid, hydroxylysine, and *γ* -aminobutyric acid (GABA).

The final data set included 21 plasma free amino acids (PFAA) that could be accurately quantified in the plasma of study participants (Table 2). The total PFAA concentration was calculated for each participant and compared between patient groups. We observed a significant reduction of the median concentration of PFAA in all patient groups when compared to healthy individuals, particularly in patients with uncomplicated malaria (Table 2 and Fig. S1). The individual levels of amino acid levels are graphically displayed in Supplementary Figs S2 and S3.

**Table 2:** Plasma concentrations of 21 free amino acids in patients and healthy controls (n = 295) from Bangladesh.

Name	Abbrev	Severe Malaria (n = 88) (µmol/L)		Uncomplicated Malaria (n = 71) (µmol/L)		SIRS/sepsis (n = 88) (µmol/L)		Healthy control (n = 48) (µmol/L)	
Alanine	Ala	549	(350 to 858)	178	(10 to 407)	445	(343 to 622)	625	(413 to 784)
Arginine	Arg	43	(33 to 51)	32	(24 to 44)	36	(28 to 45)	75	(59 to 85)
Asparagine	Asn	19.7	(16.4 to 25.2)	14.7	(11.7 to 18.5)	13.9	(11.9 to 19)	19.7	(17 to 21.9)
Citrulline	Cit	7.4	(6.9 to 8.2)	6.7	(6.4 to 7.4)	8	(6.9 to 9.1)	11.4	(10.1 to 13.3)
Cysteine	Cys	44	(19 to 59)	28	(5 to 41)	42	(33 to 56)	56	(53 to 61)
Glutamate	Gln	200	(175 to 252)	190	(148 to 228)	208	(175 to 255)	259	(239 to 284)
Glutamine	Glu	190	(116 to 305)	130	(61 to 294)	302	(147 to 434)	718	(469 to 954)
Glycine	Gly	55	(37 to 72)	26	(8 to 47)	47	(36 to 68)	82	(64 to 100)
Glycine-Proline	Gly-Pro	343	(93 to 789)	184	(38 to 361)	493	(304 to 935)	536	(425 to 700)
Histidine	His	262	(200 to 327)	144	(101 to 202)	155	(105 to 210)	290	(266 to 337)
Hydroxyproline	Hyp	8.3	(6.5 to 10.8)	5.9	(5 to 6.9)	5.5	(5 to 6.4)	7.7	(6.8 to 8.9)
Leucine	Leu	57	(49 to 72)	38	(24 to 53)	48	(37 to 63)	51	(45 to 64)
Lysine	Lys	1150	(897 to 1410)	814	(580 to 1130)	802	(516 to 1148)	1460	(1265 to 1660)
Methionine	Met	20.2	(18.1 to 22.5)	19.4	(18.1 to 21.8)	18.5	(17.3 to 19.7)	19.7	(19 to 20.7)
Ornithine	Orn	196	(106 to 377)	129	(22 to 239)	219	(94 to 333)	519	(437 to 663)
Phenylalanine	Phe	85	(65 to 109)	57	(49 to 70)	52	(40 to 68)	37	(33 to 40)
Proline	Pro	551	(417 to 696)	277	(128 to 443)	376	(274 to 477)	540	(455 to 594)
Serine	Ser	27.5	(20.8 to 34.4)	24.1	(17.9 to 33.1)	26.6	(19 to 33.3)	42	(35.5 to 48)
Tryptophan	Trp	64	(27 to 136)	36	(0 to 107)	26	(0 to 82)	266	(235 to 300)
Tyrosine	Tyr	67	(43 to 98)	34	(19 to 63)	57	(41 to 76)	91	(83 to 107)
Valine	Val	790	(603 to 968)	387	(128 to 611)	579	(446 to 799)	704	(524 to 936)
Total PFAA (mmol/L)		4.9	(3.9 to 6.2)	2.9	(1.9 to 3.7)	4.4	(3.3 to 5.6)	6.4	(5.4 to 7.4)

Data are median (IQR).

## Amino acid derangements in malaria and sepsis

**Figure 1:**
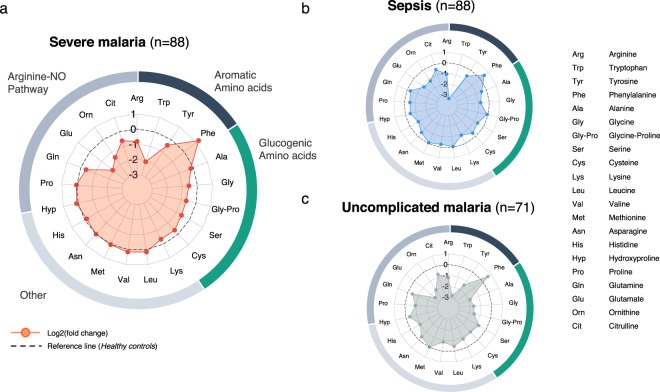
Radar plots of the plasma free amino acid profiles of study groups. (**a**) Severe falciparum malaria (n = 88). (**b**) SIRS/Sepsis (n = 71). (**c**) Uncomplicated Malaria (n = 88). The log2 normalised median fold change of each amino acid was calculated compared to the normal levels measured in healthy controls and visualised in a radar plot.

To determine the patterns of change of individual amino acids, we profiled PFAA by calculating the fold change of each amino acid compared to healthy controls and plotting the log2 normalised changes in a radar plot (Fig. 1). With the exception of phenylalanine, we observed that in each of the study groups the majority of amino acids were below normal levels.

Patients with severe malaria showed an average −23% reduction of the total concentration of PFAA. This reduction was largely due to lower concentrations of amino acids in the arginine-NO pathway, aromatic amino acids (with the exception of phenylalanine), and glucogenic amino acids.

Uncomplicated malaria was associated with a −54% reduction of the total concentration of PFAA. All amino acids, except phenylalanine, were below normal levels. Particularly metabolites from the arginine-NO pathway were low, indicating a deficiency of L-arginine and L-arginine precursors in uncomplicated malaria.

Sepsis was associated with a −32% reduction in the total PFAA concentration. A widespread reduction in amino acids was observed, particularly characterised by a reduction in tryptophan, while phenylalanine was increased.

## Association of amino acid profiles with coma, AKI, and hyperlactataemia

**Figure 2:**
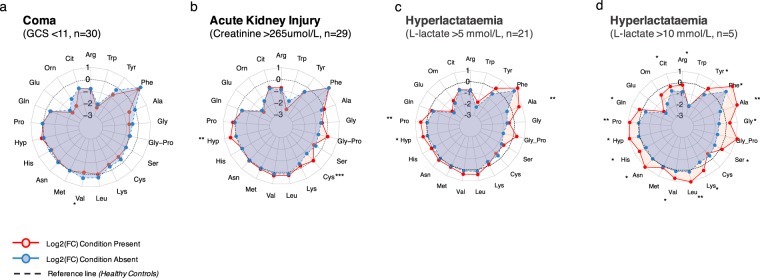
Radar plots of the plasma free amino acid profiles of major clinical syndromes of severe falciparum malaria, including coma, acute kidney injury, and hyperlactataemia. The log2 normalised median fold change of each amino acid was calculated compared to the normal levels measured in healthy controls and visualised in a radar plot.

We further analysed the amino acid profiles of 88 patients with severe malaria in relation to major clinical manifestations: coma, acute kidney injury, and hyperlactataemia (Fig. 2).

Coma, as defined by a Glasgow Coma Scale <11^24^, was observed in a total of 30 (34%) patients with severe falciparum malaria. Having coma, using a binary cut-off, did not significantly alter the plasma free amino acid profile, except for a decrease in valine (Benjamini Hochberg (BH) adjusted p-value = 0.037). We did not observe any significant differences in the concentration of phenylalanine between patients with or without cerebral malaria nor was there a significant correlation between GCS and phenylalanine (Spearman’s rho = −0.17, p = 0.13).

Acute kidney injury, defined by a serum creatinine >265 µmol/L (3 mg/dL)^24^, was found in 29 (33%) patients. Patients with acute kidney injury had significantly higher levels of hydroxyproline (BH adj. p = 0.0037) and cysteine (BH adj. p = 6.4 × 10^−4^).

Hyperlactataemia, as defined by a venous L-lactate >5 mmol/L^24^, was diagnosed in a total of 21 (23%) of patients with severe malaria. Incremental changes in venous L-lactate were associated with an increasing total concentration of plasma free amino acids (Fig. 2). L-lactate correlated with plasma alanine (Spearman’s rho = 0.57, p = 3.5 × 10^−8^), as reported earlier^8^, and phenylalanine (Spearman’s rho = 0.37, p = 4.5 × 10^−4^), and proline (Spearman’s rho = 0.39, p = 2.4 × 10^−4^). Regardless of the degree of lactic acidosis, the overall concentrations of glutamate and tryptophan remained well below the normal levels seen in healthy controls.

## Amino acid profiles associated with death

**Figure 3:**
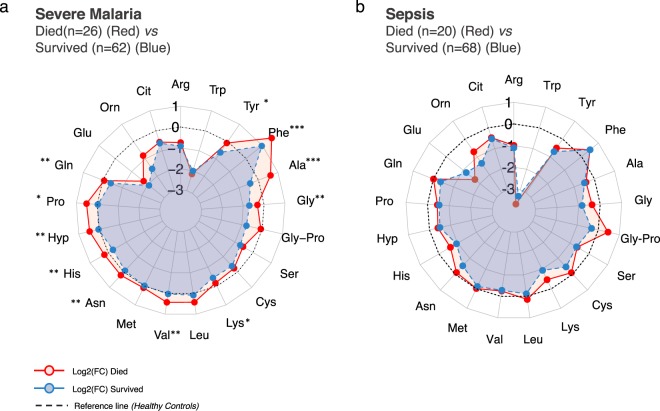
Radar plot of the plasma free amino acid profiles of patients with severe falciparum malaria or sepsis who survived their infection and in patients who died. The log2 normalised median fold change of each amino acid was calculated compared to the normal levels measured in healthy controls and visualised in a radar plot.

In patients with severe malaria, the amino acid profiles at study enrolment in patients who died compared to survivors generally showed an overall increase in the wide range of amino acids (Fig. 3). The altered amino acid profiles observed in patients who died resembled the patterns observed in patients with hyperlactataemia. A total of 10 out of 21 measured amino acids were significantly elevated in patients who died from severe malaria compared to those who survived their infection. These changes included a significant increase in plasma alanine (BH adj. p = 5 × 10^−4^), phenylalanine (BH adj. p = 5 × 10^−4^), and proline (BH adj. P = 0.011). In contrast, among patients with sepsis there were no significant differences between the levels of plasma amino acids between patients who survived and those who died.

## Determinants of total plasma amino acid levels in adults with severe falciparum malaria

We modelled the effects of L-lactate on the total concentration of PFAA using multivariate regression analysis, adjusting for factors that could explain an energy deficit or malnutrition. We wanted to assess the effect of L-lactate on the total concentration of plasma free amino acids, and used a causal inference based method including directed acyclical graphs (DAGs) (Supplementary Fig. S4). To adjust for an acute energy deficit, we included duration of illness, a measure of coma depth (GCS), and blood glucose levels (to account for hypoglycaemia). We adjusted for the total parasite biomass by including log10 normalised plasma *Pf*HRP2. Renal elimination was adjusted for by including serum creatinine. We accounted for pre-existing malnutrition by including body mass index (BMI).

Based on our multivariate linear regression model, and if our assumptions are correct, coma depth (GCS) and L-lactate are independent predictors of total PFAA (Table 3). We observed a strong effect of L-lactate on the total PFAA concentrations in patients with severe falciparum malaria, where incremental levels of L-lactate were significantly associated with increased total concentrations of PFAA. Patients who were in a deeper coma were also more likely to have higher total PFAA levels.

**Table 3:** Multivariate linear regression model to predict changes in the total concentration of plasma free amino acids (µmol/L) in patients with severe malaria (n = 88) in Bangladesh.

Variable	Estimate	SE	p-value	
BMI	68.8	62.3	0.273	
Duration of illness*	2.8	48.2	0.953	
Coma depth^†^	−125.3	55.9	0.028	*
*Pf*HRP2^‡^ (ng/dL)	−598.7	505.1	0.240	
Creatinine (µmol/L)	1.6	1.1	0.148	
Glucose (mmol/L)	61.8	58.3	0.293	
L-lactate (mmol/L)	342.3	72.6	1.3^−5^	***

Regression coefficients (estimates) and standard errors (SE) are reported. BMI = body mass index. PfHRP2 = *Plasmodium falciparum* Histidine Rich Protein 2. *Duration of illness before enrolment in days. ^†^Depth of coma was assessed by the Glasgow Coma Scale (3–15). ^‡^Log10 Normalized.

## Discussion

In this prospective observational cohort of patients with malaria and sepsis we investigated the patterns and determinants of plasma free amino acid derangements during severe infection. Using HPLC-MRM-MS we quantified 21 free amino acids in the plasma of 295 study participants. All patient groups showed a general reduction in the total concentration of amino acids on enrolment compared to normal levels observed in local healthy controls. Patients with uncomplicated malaria showed the largest decrease of PFAA (−54%). We analysed the amino acid profiles in relation to clinical manifestations and outcome in patients with severe malaria. Although the total concentration of PFAAs was on average decreased compared to normal levels (on average −23%), hyperlactataemia was associated with widespread increase, likely because this reflects tissue hypoxia. We used a multivariate regression model to determine the effect of L-lactate on the plasma concentration of free amino acids in patients with severe falciparum malaria and observed that after adjusting for potential confounders, L-lactate likely caused by tissue hypoxia remained a significant predictor of an increased total concentration of PFAA.

These observations confirm previous findings of amino acid derangements in patients with severe falciparum malaria, including derangements in L-arginine, alanine, and phenylalanine metabolism, however, we show they are part of a widespread pattern of amino acid dysregulation. Concentrations of PFAA were generally decreased in patients with severe malaria, but also in sepsis and uncomplicated malaria. Previously described individual amino acid derangements are in part explained by this, and are therefore less likely to be specific for certain presenting syndromes. In cases with malaria and sepsis, we observe amino acids are depleted from plasma, which is likely due to increased hepatic amino acid extraction from blood, as has been shown before in patients with inflammation and sepsis^17,19,21^. Although there is on average a negative nitrogen balance in patients with severe falciparum malaria, we observe that L-lactate, as a marker for tissue hypoxia, is associated with a widespread increase in nearly all amino acids, probably due to increased muscle protein breakdown. The relationship between increased plasma L-lactate and raised PFAA may also be related to impaired liver blood flow with hepatic tissue dysoxia^25,26^, leading to a reduction of hepatic PFAA extracted from plasma.

Our data are consistent with previous reports of L-arginine deficiency in patients with severe and uncomplicated malaria^5,6,27–29^. Previously, L-arginine deficiency was attributed to increased metabolism of L-arginine following intravascular nitric oxide depletion^30^ and by arginases from host^29^ or parasite^31,32^. It has recently been proposed that L-arginine deficiency might arise from low bioavailability of its precursors^33^. We measured two L-arginine precursors, ornithine and citrulline, and observed both were significantly depleted in uncomplicated malaria. Our findings support the hypothesis that low-bioavailability of precursors contributes to L-arginine deficiency in malaria, early during the disease process, and probably because of a overall negative nitrogen balance.

Current data support previous findings of a correlation between L-lactate and plasma alanine in severe falciparum malaria^8^. Muscle protein catabolism increases nitrogen levels during tissue hypoxia and anaerobic glycolysis. Nitrogen is shuttled to the liver through the Cahill cycle in the form of alanine, where it is utilised in gluconeogenesis. However, high levels of L-lactate may reduce the hepatic disposal alanine and of other glucogenic amino acids^8,34^, which likely explains their strong correlation.

Our data confirm hyperphenylalaninaemia in patients with severe malaria, as described previously^9–12^. Similar to prior studies, we also note that patients with sepsis and uncomplicated malaria have significantly increased plasma levels of phenylalanine. Previous studies have investigated if there may be a link between hyperphenylalaninaemia and coma^9–12^. Hyperphenylalaninaemia has been attributed to reduced activity from phenylalanine-hydroxylase due to a deficiency of its co-factor tetrahydrobiopterin^9,11,12,33^ which affects the metabolism of aromatic amino acids (Arg, Trp, Phe, Tyr) and the biosynthesis of neurotransmitters from the phenylalanine/tyrosine pathways. Unfortunately we were unable to measure biopterins in this study and therefore cannot conclude a role for aromatic amino acid co-factor deficiencies in our study population. However, increased phenylalanine:tyrosine ratios among patients with malaria or sepsis were suggestive of reduced conversion of phenylalanine to tyrosine (Supplementary Fig. S5). Among patients with severe malaria, we did not observe a relationship between phenylalanine and coma depth (Spearman’s rho = −0.17, p = 0.13), as shown previously in both adult and pediatric severe malaria^10^. Although it is not completely clear what causes hyperphenylalaninaemia in severe falciparum malaria, it is not specific to malaria alone and also occurs in sepsis.

In conclusion, the concentrations of plasma free amino acid are decreased in patients with severe malaria, sepsis and uncomplicated malaria. Previously described individual amino acid derangements are in part explained by this, and less likely specific for certain presenting syndromes. Hyperlactataemia increased the total amino acid concentration in patients with severe malaria, likely because this reflects tissue hypoxia.

## Methods and Materials

### Ethical statement

This was a sub-study of an observational study protocol that was reviewed and approved by the local ethics review board, the Chittagong Medical College Ethics Committee, Bangladesh, and the Oxford Tropical Research Ethics Committee (OxTREC), in the United Kingdom. The trial was registered at *ClinicalTrials.gov* (Trial number: NCT02451904). All research staff involved with providing patient care received training according to the Guidelines for Good Clinical Practice; the studies were conducted according to the Declaration of Helsinki^35,36^. All participants provided written informed consent prior to enrolment. Individual written informed consent was obtained from the patient or from their attending relatives if they were unable to give consent themselves due to the severity of their illness.

### Study Design

A prospective observational case-control study in Chittagong Medical College Hospital, Bangladesh. The recruitment phase of the study spanned four wet-seasons and was from May-September 2014 and May-September 2017.

### Study participants and procedures

We consecutively enrolled male and female adults (>12 years) with falciparum malaria and patients with sepsis. Patients with a blood slide positive for asexual blood stages of *P. falciparum* (including mixed infection with non-falciparum species) were eligible to participate in the malaria study group. Severe and uncomplicated falciparum malaria were strictly defined according the WHO criteria for severe malaria^24^, modified by Hien *et al*.^37^. Patients who had a confirmed negative blood slide for malaria were assessed for eligibility in the sepsis study group and consecutively enrolled. A minority of patients had already received a first dose of antimalarials prior to the study, however, cases were excluded if they received antimalarial therapy >24 hours before enrolment. Healthy controls were required to have a blood slide negative for malaria, normal vital signs (no hypertension). Healthy controls were excluded if they were pregnant, had a chronic illness, took medication in the prior two months before enrolment.

On enrolment, we took a medical history, a medication history, assessed vital signs, performed a physical examination and drew blood samples for blood gas analysis and amino acid analysis. Participants were not required to be fasting at the time of blood draw. Patients were followed daily until hospital discharge or in-hospital death. Laboratory assessments included haematology, which was done in a quality controlled external laboratory (Chevron Clinical Lab, Chittagong, Bangladesh). We used iStat analysers (Abbott, Chicago, Illinois) for biochemistry (Chem8 + cartridge) and blood gas and L-lactate analysis (CG4 + cartridge).

### HPLC-MRM-MS

Blood for amino acid analysis was drawn from a fresh catheter in the forearm in non-fasting adults immediately following study enrolment and usually before a first dose of antimalarials was provided. Blood samples was collected in lithium-heparin tubes that were immediately centrifuged and flash-frozen in liquid-nitrogen. Samples were shipped on dry ice and stored at −80 °C. In brief, amino acids were extracted by solid-phase extraction using sorbent tips (Phenomenex EZ:faast™), as described previously^38^. Plasma free amino acids (PFAA) were separated on an AAA-MS™ column (250 × 3.0 mm, Phenomenex, Torrance, California) placed in a Thermo LC system. Mass spectrometry with multiple reaction monitoring was done in positive ion mode with electrospray ionization on an ABSciex API 5000 Triple Quadrupole LC/MS/MS Mass Spectrometer. Inter-assay variation scores were determined for three amino acid standards and the individual amino acids. Concentrations were based on interpolation on a linear standard curve. We excluded amino acids if they could be measured in less than 50 cases. The final amino acid data set is publicly available on GitHub, found at: https://github.com/Stije/PFAA.

### Statistical analysis

Data analysis was carried out in R statistical software (V3.3.3). Baseline characteristics in the study groups and the plasma concentration of free amino acids are all presented as median with inter quartile ranges (IQR), unless stated otherwise. Categorical data were reported as number and proportions. Significance was estimated for multiple study groups (>2) using Kruskal-Wallis tests and corrected by a Benjamini-Hochberg procedure, where appropriate. Significance was estimated for two study groups by Mann-Whitney U tests. Associations were tested for using Spearman’s rank correlation. The sum of all measured amino acids (per individual) was calculated to determine the total plasma concentration of free amino acids per individual. Amino acid profiles were visualised in radar plots using the’fmsb’ package in R. Radar plots contained normalised amino acid concentrations (the log2 median fold change of patients compared to healthy controls).

We wanted to determine the potential effects of tissue hypoxia on the total concentration of PFAA, and used directed acyclic graphs (DAGs) as a causal inference framework (Supplementary Fig. S4). This method requires that the direct causal effects between variables of interest are specified based on a subjective exercise, which has the benefit of clearly displaying the assumptions underlying the results. We based our DAG on the assumption that the total concentration of PFAA reflects a balance between malnutrition, an energy deficit and tissue hypoxia. The potential mechanisms are depicted in the DAG. We used a multivariate linear regression model to characterise the relationship between tissue hypoxia and the total measured PFAA. The standard linear regression was fitted using the function *glm* in R.

## Supplementary information

Supplementary Information
