# Supplementary material for: Amino acid derangements in adults with severe falciparum malaria
Source: Sci Rep. 2019 Apr 29;9:6602. doi: 10.1038/s41598-019-43044-6 (PMC6488658; doi:10.1038/s41598-019-43044-6)
Supplement: Supplementary file 1 — Supplementary Information [file 41598_2019_43044_MOESM1_ESM.pdf]

Supplementary Information

Amino acid derangements in adults with severe falciparum malaria

Stije J. Leopold[1,2], Siribha Apinan[1], Aniruddha Ghose[2], Hugh W. Kingston[1], Katherine A. Plewes[1,2], Amir Hossain[3], Asok Kumar Dutta[3], Sujat Paul[3], Anupam Barua[3], Abdus Sattar[3], Nicholas P.J. Day[1,2], Joel Tarning[1,2], Markus Winterberg[1,2], Nicholas J. White[1,2], and Arjen M. Dondorp[1,2,*]

[1]Mahidol-Oxford Tropical Medicine Research Unit, Faculty of Tropical Medicine, Mahidol University, Bangkok, Thailand
[2]Centre for Tropical Medicine and Global Health, Nuffield Department of Medicine, University of Oxford, United Kingdom
[3]Department of Internal Medicine, Chittagong Medical College Hospital, Chittagong, Bangladesh
[*]arjen@tropmedres.ac

Supplementary Materials

Supplementary Fig. S1. Total plasma free amino acid concentrations in adult patients and healthy controls from Bangladesh.

Supplementary Fig. S2a. Individual plasma free amino acids in adult patients and healthy controls from Bangladesh. (Part A).

Supplementary Fig. S2b. Individual plasma free amino acids in adult patients and healthy controls from Bangladesh. (Part B).

Supplementary Fig. S3. Directed acyclic graph for the causal effect identification of L-lactate on the total concentration of plasma free amino acids in adults with severe falciparum malaria.

Supplementary Fig. S4. Phenylalanine:Tyrosine ratio in adult patients with severe falciparum malaria from Bangladesh.

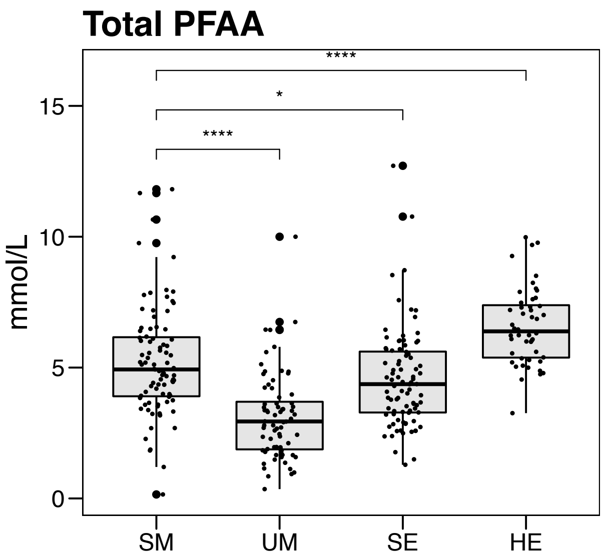

Figure 1. Total plasma free amino acid concentrations in adult patients and healthy controls from Bangladesh (mmol/L). Amino acids were measured by UHPLC-MRM-MS in adults with severe malaria (n=88), uncomplicated malaria (n=71), Sepsis (n=88), and healthy local controls (n=48). Boxplots with medians and inter quartile ranges are shown. Mann-Whitney U test: $p \leq 0.05$ = '*', $p \leq 0.01$ = '**', $p \leq 0.001$ = '***', $p \leq 0.0001$ = '****'.

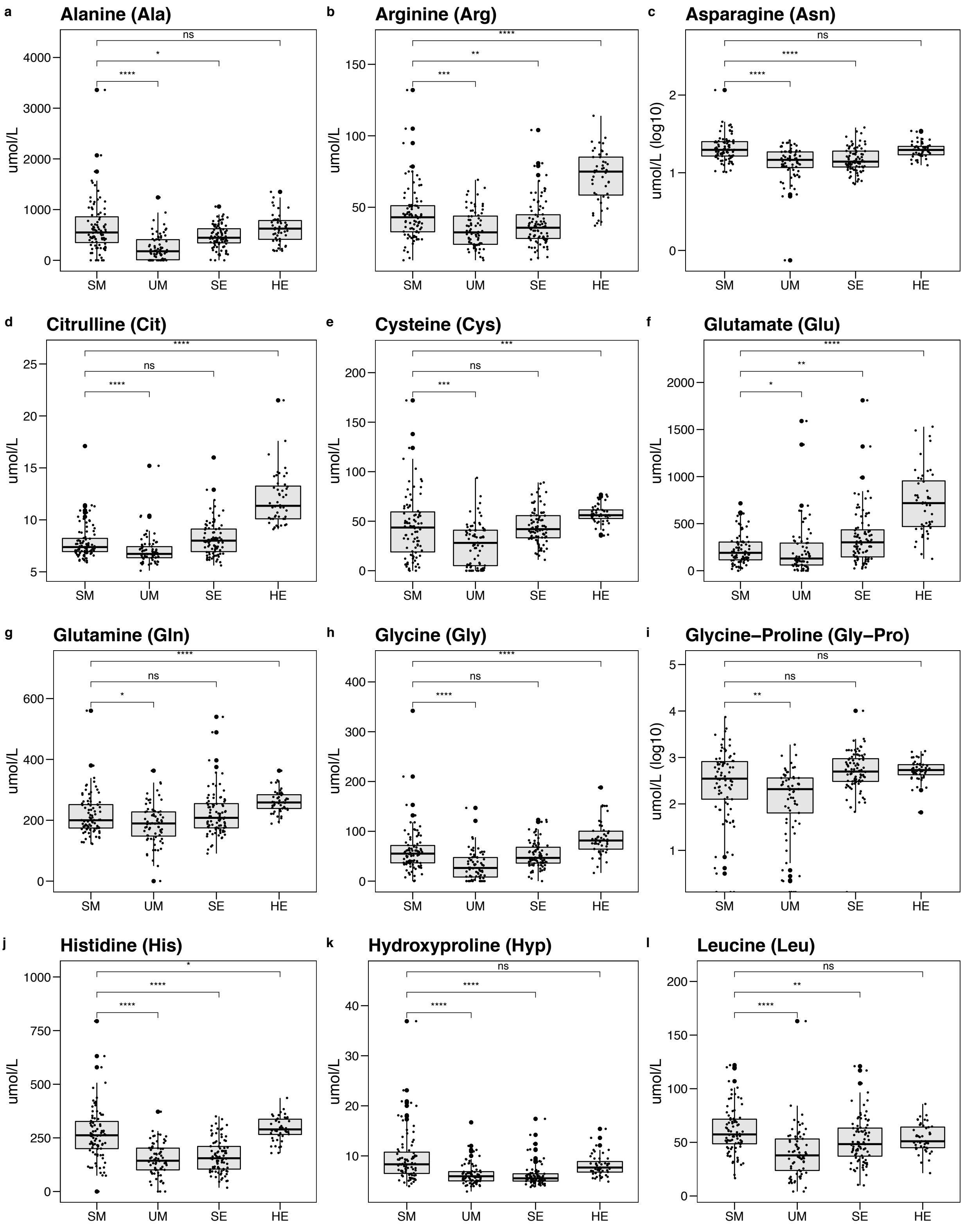

Figure 2. Individual plasma free amino acids in adult patients and healthy controls from Bangladesh; (µmol/L) (Part A). Amino acids were measured by UHPLC-MRM-MS in plasma of study participants (n=295) from Bangladesh. Boxplots with medians and inter quartile ranges are shown. Mann-Whitney U test: $p \leq 0.05$ = '*', $p \leq 0.01$ = '**', $p \leq 0.001$ = '***', $p \leq 0.0001$ = '****'.

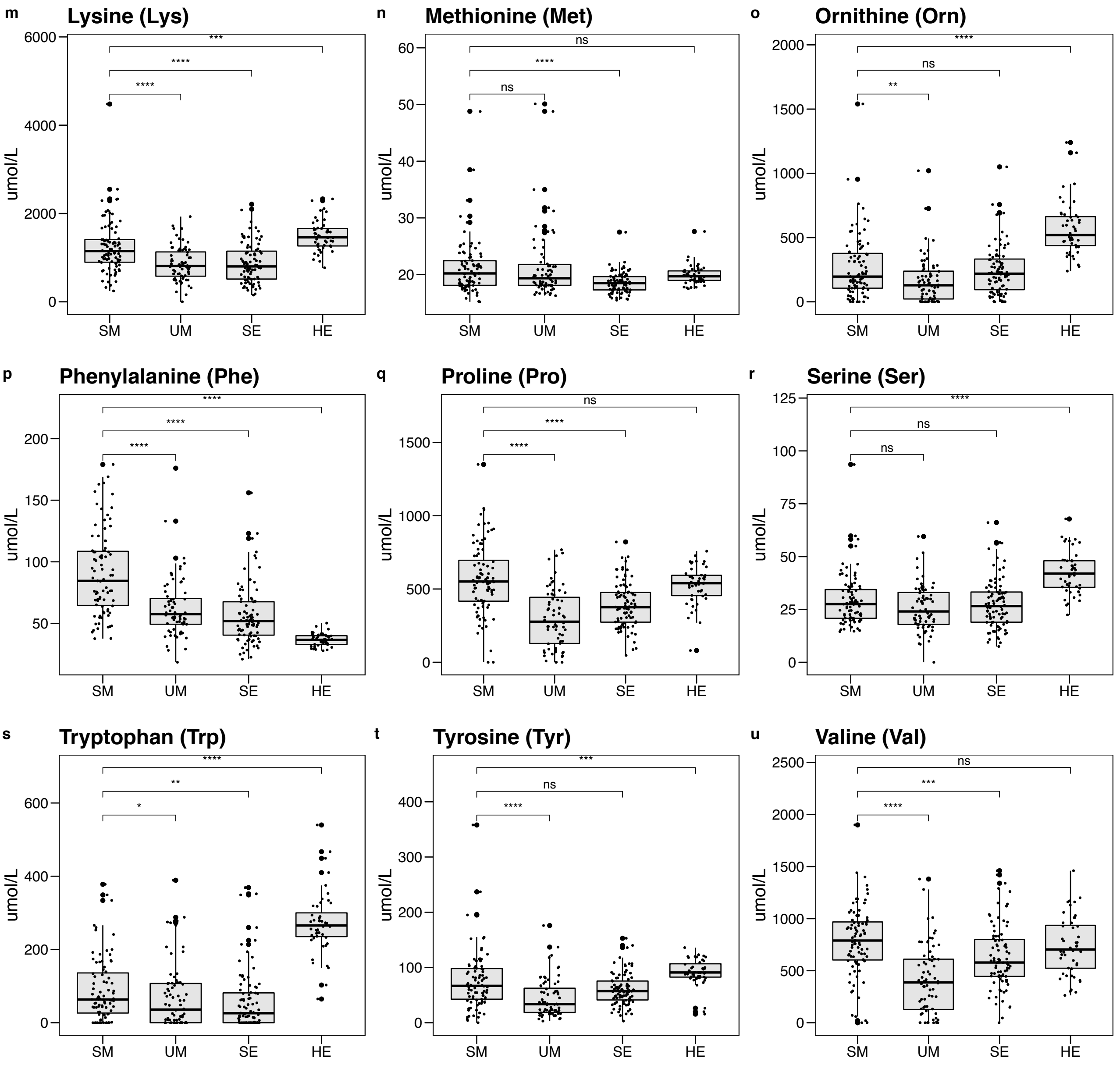

Figure 3. Individual plasma free amino acids in adult patients and healthy controls from Bangladesh; (µmol/L) (Part B). Amino acids were measured by UHPLC-MRM-MS in plasma of study participants (n=295) from Bangladesh. Boxplots with medians and inter quartile ranges are shown. Mann-Whitney U test: $p \leq 0.05$ = '*', $p \leq 0.01$ = '**', $p \leq 0.001$ = '***', $p \leq 0.0001$ = '****'.

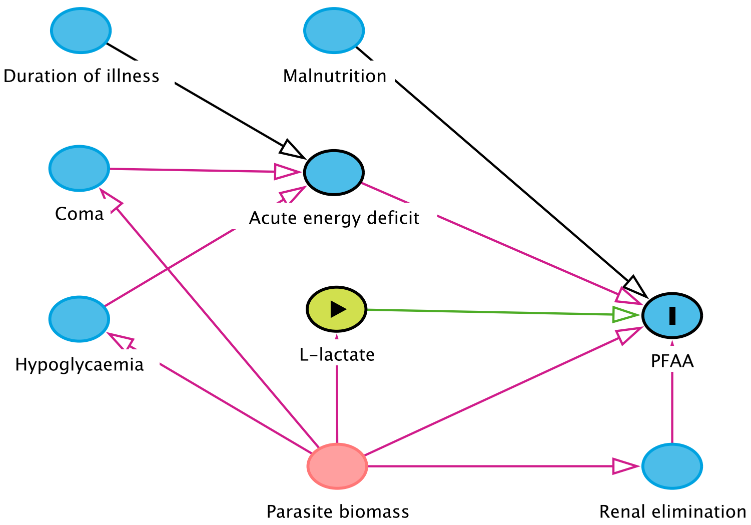

Figure 4. Directed acyclic graph for the causal effect identification of L-lactate on the total concentration of plasma free amino acids in adults with severe falciparum malaria. L-lactate (depicted using the green vertex) was set as the 'exposure' variable of interest, and 'PFAA' the outcome variable. Each factor is denoted by a vertex in the graph. The direct effect of L-lactate (tissue hypoxia) on PFAA (green pathway) can be derived with minimal sufficient adjustment sets by adjustment for parasite biomass. (reproducible DAG can be found at dagitty.net/mlGs7Fc).

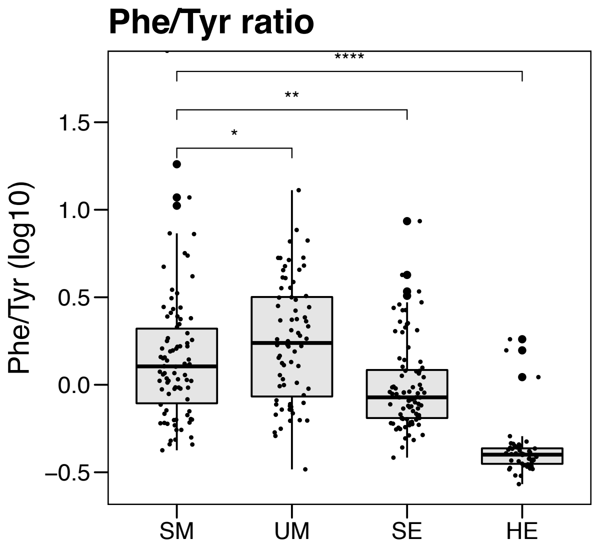

Figure 5. **Phenylalanine:Tyrosine ratio in adult patients with severe falciparum malaria from Bangladesh.** Boxplots with medians and inter quartile ranges are shown. Mann-Whitney U test: $p \leq 0.05$ = '*', $p \leq 0.01$ = '**', $p \leq 0.001$ = '***', $p \leq 0.0001$ = '****'.
